# Greater angiogenic and immunoregulatory potency of bFGF and 5-aza-2ʹ-deoxycytidine pre-treated menstrual blood stem cells in compare to bone marrow stem cells in rat model of myocardial infarction

**DOI:** 10.1186/s12872-022-03032-7

**Published:** 2022-12-31

**Authors:** Mahmood Manshori, Somaieh Kazemnejad, Nasim Naderi, Maryam Darzi, Nahid Aboutaleb, Hannaneh Golshahi

**Affiliations:** 1 Nanobiotechnology Research Center, Avicenna Research Institute, ACECR, Tehran, Iran; 2 Rajaie Cardiovascular Medical and Research Center, Iran University of Medical Sciences, Tehran, Iran; 3 Physiology Research Center, Iran University of Medical Sciences, Tehran, Iran; 4 Department of Physiology, Faculty of Medicine, Iran University of Medical Sciences, Tehran, Iran

**Keywords:** Myocardial infarction, Menstrual blood stem cells, Bone marrow stem cells, Basic fibroblast growth factor, 5-Azacytidine

## Abstract

**Background:**

This study is designed to compare the menstrual blood stem cells (MenSCs) and bone marrow stem cells (BMSCs)-secreted factors with or without pre-treatment regimen using basic fibroblast growth factor (bFGF) and 5-aza-2ʹ-deoxycytidine (5-aza) and also regenerative capacity of pre-treated MenSCs and/or BMSCs in a rat model of myocardial infarction (MI).

**Methods:**

BMSCs and MenSCs were pre-treated with bFGF and 5-aza for 48 h and we compared the paracrine activity by western blotting. Furthermore, MI model was created and the animals were divided into sham, MI, pre-treated BMSCs, and pre-treated MenSCs groups. The stem cells were administrated via tail vain. 35 days post-MI, serum and tissue were harvested for further investigations.

**Results:**

Following pre-treatment, vascular endothelium growth factor, hypoxia-inducible factor-1, stromal cell-derived factor-1, and hepatocyte growth factor were significantly increased in secretome of MenSCs in compared to BMSCs. Moreover, systemic administration of pre-treated MenSCs, leaded to improvement of cardiac function, preservation of myocardium from further subsequent injuries, promotion the angiogenesis, and reduction the level of NF-κB expression in compared to the pre-treated BMSCs. Also, pre-treated MenSCs administration significantly decreased the serum level of Interleukin 1 beta (IL-1β) in compared to the pre-treated BMSCs and MI groups.

**Conclusions:**

bFGF and 5-aza pre-treated MenSCs offer superior cardioprotection compare to bFGF and 5-aza pre-treated BMSCs following MI.

**Supplementary Information:**

The online version contains supplementary material available at 10.1186/s12872-022-03032-7.

## Background

Ischemic heart disease remains one of the leading cause of mortality worldwide. Despite noticeable improvement in the treatment and management of this condition, the prognosis for patients with heart failure (HF) remains poor. Over the past decade, cell-based therapy has emerged as a potential new alternative therapeutic approach for the regeneration of the ischemic myocardium after myocardial infarction (MI) and preventing subsequent HF [1]. Stem cell transplantation can preserve and/or regenerate functional myocardial tissue, enhance tissue perfusion, contribute to neoangiogenesis and immunomodulation by several mechanisms including differentiation, stimulate growth of residual myocytes via secretion signalling factor, mediate the microenvironment, promote the resident stem cell, induction of cell fusion between transplanted stem cells and native cardiomyocytes, and interactions and cross talk among endothelial cells, immune cells, and cardiomyocytes [2–5].

Bone marrow stem cells (BMSCs) are one of the most common types of stem cells used in cell therapy. Some studies have been reported the improvement of the heart function after BMSCs transplantation to the myocardium [6, 7], however some issues including limited availability, invasive and painful sample collection procedure, and low proliferation capacity limit the applicability of BMSCs for clinical transplantation [8, 9].

Menstrual blood (MB) is a new and interesting source of stem cells. MenSCs possess noticeable advantages such as abundance, easy and non-invasive extraction and isolation process, high proliferative rate, painless procedures, and multilineage differentiation potency [9, 10]. However, researchers have been recognized menstrual blood stem cells (MenSCs) as mesenchymal stem cells (MSCs), this kind of stem cell also displays embryonic stem cell surface marker such as OCT-4 [11]. MenSCs exhibited higher proliferation rate compared to BMSCs [12]. Also researches showed that various factors such as angiopoietin-2 (ANG-2), vascular endothelial growth factor (VEGF), hepatocyte growth factor (HGF), matrix metalloproteases (MMPs), and fibroblast growth factor (FGF) released in conditioned medium (CM) of MenSCs, may be effective in the repairing of damaged tissue [12, 13].

At the MI site, the stem cells are challenged with a poor microenvironment, which results in low engraftment rate, weak viability, and poor proliferative abilities; therefore limiting the effectiveness of the therapy [14]. There is growing evidence that in vitro pre-treatment strategies can increase their therapeutic potency and could augment the infarction site recovery by enhancing survival, engraftment, and secretory properties [15–17].

5-aza-2′-deoxycytidine (5-aza) as a DNA methyl transferases inhibitor, increases the efficiency of stem cell through epigenetic modifications [18]. 5-aza has also been reported to increase immune suppressive effects and migration of MSCs [19]. Basic fibroblast growth factor (bFGF), is considered as a potential conductor required to support the effect of 5-aza in regulating myocardial differentiation [20]. bFGF, as a heparin binding growth factor, can regulate cell proliferation and migration [21, 22]. It also suppresses inflammation, promotes angiogenesis, and as a multipotent stimulus, is important for tissue regeneration [23]. It has been demonstrated that the pre-treatment of Sca-1^+^ cardiac stem cells with bFGF enhanced their targeting to the infarct site as well as improved angiogenesis [24].

In the present study; firstly, we compared the MenSCs and BMSCs-secreted factors with or without pre-treatment regimen using bFGF and 5-aza; secondly, we evaluated regenerative capacity of bFGF and 5-aza pre-treated MenSCs and/or BMSCs in a rat model of MI.

## Materials and methods

### Isolation and culture of MenSCs and BMSCs

Menstrual bloods were obtained from five healthy female volunteers with mean age of 25–35 years in the second day of menstruation by sterile Diva cup (Diva International). Isolation and culture of stem cells from MB were previously described by our group [25]. Briefly, the contents of Diva cup along with 2.5 μg/mL fungizone (GIBCO), 100 μg/mL streptomycin, 100 U/mL penicillin (Sigma-Aldrich) and 0.5 mM ethylenediaminetetraacetic acid (EDTA) in phosphate buffered saline (PBS) without Ca^2+^ or Mg^2+^ were transferred into the falcon tube. MB derived mononuclear cells were separated by a density gradient centrifugation using Ficoll-Paque (GE Healthcare). MenSCs cultured in Dulbecco's Modified Eagle Medium/Nutrient Mixture F-12 (DMEM-F12) medium (GIBCO) with 10% FBS at 37 °C with 5% CO_2_ and saturated humidity. After one-day, removal of non-adherent cells was performed, and the culture of adherent cells continued until 70–80% confluency. Adherent cells were detached using trypsin (Gibco, UK) and EDTA suspended in PBS.

BMSCs were separated from bone marrow aspirates (5–10 mL) of five healthy female donors aged 18–30 years. The samples were isolated from iliac crests at the Bone Marrow Transplantation Center, Shariati Hospital, Tehran University of Medical Sciences. The isolation procedure of BMSCs was performed using a combination of density gradient centrifugation and plastic adherence as described in our previous study [26, 27]. All experiments were carried out on MenSCs and BMSCs at passages 2–4.

### Identification of cultured MenSCs and BMSCs by flow cytometry

Evaluation of the expression of mesenchymal stem cell surface markers (CD73 and CD44), embryonic stem cell surface marker (OCT-4), and hematopoietic cell marker (CD45) was done by flow cytometric analysis as described previously [28]. Cells (10^5^ cells/100 μl) were gently washed in PBS containing 2% FBS and incubated separately with PE-conjugated mouse anti-human CD73 (561014; BD Pharmingen), CD44 (550989; BD Pharmingen), and CD45 (560975; BD Pharmingen) in darkness for at least 40 min at 4 °C. Evaluation of OCT-4 expression was analysed using indirect intracellular flow cytometry. The cells were permeabilized with 0.1% saponin. Then primary rabbit antihuman OCT-4 antibody (ab19857, Abcam) was added for 40 min and then incubated with FITC-conjugated goat anti-rabbit Ig (Sigma-Aldrich) for 30 min. As negative controls, isotype IgG (555748; BD Pharmingen) was used. Afterward, cells were washed twice with PBS-FBS, fixed in 1% formaldehyde solution, and analyzed by a flow cytometer (Partec GmbH).

### Pre-treatment of MenSCs and BMSCs with 5-aza and bFGF

MenSCs and BMSCs were cultured and expanded under normoxic condition (O_2_: 20% CO_2_: 5%). When cells reached 80% confluency, they were treated with serum-free DMEM consisting 10 μM 5-aza and 10 ng/mL bFGF. After 48 h, CM was collected from monolayer culture of 2 × 10^6^ MenSCs and BMSCs.

### Western blot analysis

The expression level of VEGF, HGF, Hypoxia-inducible factor 1-alpha (HIF-1α), Interleukin 1 beta (IL-1β), ANG-1, ANG-2, and Stromal cell-derived factor-1 (SDF-1) in MenSCs and BMSCs derived CM was evaluated before and after pre-treatment with bFGF and 5-aza by western blotting. Western blot analyses were performed as previously described with some modifications [29]. CM was centrifuged at 10 000 rpm for 20 min at 4 °C. Protein concentration was determined by the Bradford protein Quantification kit according to the manufacturer's instructions. The cells lysates were mixed with the equal volume of 2X Laemmli sample buffer. Lysates (15 μg) were separated by SDS-PAGE and subsequently transferred to a 0.2 μm Immune-Blot™ polyvinylidene difluoride (PVDF) membrane (162-017777; Bio-Rad Laboratories). The membranes were blocked with 5% BSA (A-7888; Sigma Aldrich) in 0.1% Tween 20 for 1 h. Then, the membranes were incubated with anti-SDF-1 (ab155090, Abcam), anti- HGF (ab178395, Abcam), anti-VEGF (ab46154, Abcam), anti- Ang-1 (ab183701, Abcam), anti- Ang-2 (ab155106, Abcam), anti-HIF-1α (ab179483, Abcam), anti-IL-1β (ab216995, Abcam), and anti-beta actin-loading control antibodies (ab8227; Abcam) for 1 h at room temperature (RT). Subsequently, membranes were washed with TBST (Tris-buffered saline with 0.1% Tween® 20 Detergent), and incubated with goat anti-rabbit IgG H&L (HRP) (ab6721; Abcam). The membranes were then incubated with enhanced chemiluminescence (ECL) for two min. Protein expression was normalized to β-actin. Densitometry of protein bands was performed using the Gel Analyzer Version 2010a software (NIH, USA), such that, the percentage area under the curve of each band was divided by the percentage area under the curve of its corresponding actin band, and then calculated values were compared among groups.

### In vivo* modelling*

45 male Wistar rats (10–12 weeks old; weight, 300–350 g) were obtained from the animal laboratory of Iran University of Medical Sciences. They were housed in polycarbonate cages inside a well-ventilated room kept on a 12-h light/12-h dark cycle at an average temperature of 24 ± 2 °C, with 50 ± 10% relative humidity, with a standardized regular diet and water ad libitum.

### Induction of MI

The animals were anesthetized with ketamine (75 mg/kg) and xylazine (5 mg/kg) intraperitoneally and intubated and ventilated by a rodent ventilator (tidal volume 2–3 mL, respiratory rate 65–70 per min). After a left thoracotomy at the fourth intercostal space; left anterior descending (LAD) artery coronary was permanently ligated (about 2 mm distal from the tip of the left auricle) using a 6-0 prolene suture for induction of myocardial infarction. MI was successfully approved by the development of a pale colour in the myocardial surface distal to the suture and dyskinesia of the anterior wall [30]. After surgery, the ventilator was removed, and the animals monitored until full recovery. Sham-operated rats (n = 8) experienced a similar procedure without coronary artery ligation. Seven days after induction of MI, the surviving animals were narcotized, and their Fractional shortening (FS) and ejection fraction (EF) were evaluated. Then, the rats were randomly divided into three experimented groups (n = 8): (1) MI: 200 μl PBS; (2) bFGF, 5-aza pre-treated MenSCs: 2 × 10^6^ MenSCs suspended in 200 μl MenSCs-CM; (3) bFGF, 5-aza pre-treated BMSCs: 2 × 10^6^ BMSCs suspended in 200 μl BMSCs-CM. Seven days after MI induction, the rats in group 2–3 received their treatments via tail-vein. Postoperative care was preserved utilizing analgesia and hemodynamic monitoring for 48 h.

### Echocardiography

Echocardiographic evaluation was performed under light anaesthesia by ketamine and xylazine on days 7 and 35 post-surgery. Transthoracic two-dimensional (2D) guided M-mode echocardiography (General Electric-Vingmed Ultrasound, Horten Norway) was done using a 10 MHz electronic linear transducer. Cardiac parameters such as left ventricle internal diameter in diastole (LVIDd) and left ventricle internal diameter in systole (LVIDs) were obtained after 3–5 consecutive heart cycles. FS and EF were calculated according to the formula respectively: [LVIDd − LVIDs)/LVIDd] × 100 and (LVIDd2 − LVIDs2)/LVIDd2 × 100. The ΔFS (% change in FS for each rat) and ΔEF (% change in EF for each rat) were also calculated.

### Histological examination

35 days after the beginning of the process, animals were euthanized, and heart tissues were collected and fixed with 10% neutral buffered formalin (NBF). After dehydration and embedding in buffered paraffin, the samples were sectioned at 5 μm thickness, and stained with haematoxylin and eosin (H&E) and Masson’s trichrome. The infarct size was expressed as the percent ratio (%) of the infarct area, divided by the whole left ventricular (LV) area.

### Immunohistochemical assay of NF-κB expression

After deparaffinization and rehydration of the slides, the tissue sections were treated with 3% H_2_O_2_ (in methanol) for 10 min. After washing with distilled water for 2 min (3X), the slides were placed in citrate buffer (0.01 M, pH = 6) and heated to boiling for 10 min. The slides were allowed to cool at RT. 2% bovine serum albumin mixed in normal sheep serum (Avicenna Research Institute, Tehran, Iran) was used as blocking agent. After that, the slides were incubated with a primary antibody for NF-κB, (ab16502, Abcam, 1:500) overnight at 4 °C. After washing, slides were incubated with secondary antibody (K5007, DAKO) for 1 h at RT. To visualize immunoreactivity, 3, 3’-Diaminobenzidine (DAB) made up with substrate buffer (K5007, DAKO), was added to slides. Finally, the sections were counterstained with Mayer’s haematoxylin (Sigma) for 2 min, dehydrate, and mounted. The percent of NF-κB expression was quantitatively analysed by ImageJ software (ImageJ, NIH, Bethesda, MD, USA). The slides were examined using a microscope (Olympus BX51) connected to a digital camera (Olympus, DP71) by a veterinary anatomic pathologist.

### Tracking of engraftment of injected stem cells using immunohistochemistry

Deparaffinized sections were rinsed with Tris-buffered saline (TBS). The antigen retrieval step was done by microwave heating base using EDTA buffer (pH = 9) and then endogenous biotin was blocked with Biotin-Blocking System (X0590, Dako). For blocking, normal mouse serum (Avicenna Research Institute, Tehran, Iran) was used. To tracking of injected stem cells, the sections were incubated with the mouse anti-human anti-mitochondrial antibody (MAB1273B, Merk, 1:150) overnight at 4 °C. The continuation of the process was the same as the method mentioned above.

### Evaluation of angiogenesis

The sections were incubated with anti-cluster of differentiation 31 (CD31) primary antibody (orb10314; Biorbyt, 1:100) overnight at 4 °C. After washing, slides were incubated with the secondary antibody (orb688925; Biorbyt, 1:150) for 2 h at 37 °C. Afterward, the slides were washed and incubated with DAPI (Sigma-Aldrich, D9542) for nuclear staining at RT. Vascular density was quantified by the counting of stained capillary structures in 5 randomly high-power fields (HPF) per sample in the infarct border zone.

### ELISA assay

Blood samples were collected into non-heparinized tubes and centrifuged at 3000 g for 10 min, and the obtained serum was stored at − 80 °C. The amount of Interleukin-6 (IL-6) (R6000B, R&D Systems), tumour necrosis factor-α (TNF-α) (RTA00, R&D Systems), and IL-1β (RLB00, R&D Systems) in serum samples were measured with an enzyme-linked immunosorbent assay (ELISA). In brief, known concentrations of recombinant rat IL-6, TNF-α, or IL-1β and the experimental samples were added and incubated in polystyrene microtiter plates coated with an antibody against the appointed cytokine, followed by incubation with an enzyme-linked polyclonal antibody directed to the cytokine. Next, a substrate solution for the enzyme was added, and the colour development was stopped by adding 2N H2SO4. The absorbance was measured with a microtiter plate spectrophotometer. The amount of IL-6, TNF-α, and IL-1β in each sample was determined from a standard curve generated in each assay and expressed as pictograms per millilitre.

### Statistical analysis

All data were expressed as mean ± SD. For comparing the differences among groups, one-way analysis of variance was used. Statistical analyses were performed by SPSS 20.0 software (IBM Corp., Armonk, NY, http://www.ibm.com). *P* value < 0.05 was accepted to be a statistically significant difference.

## Results

### Phenotypes of isolated MenSCs and BMSCs

**Fig. 1:**
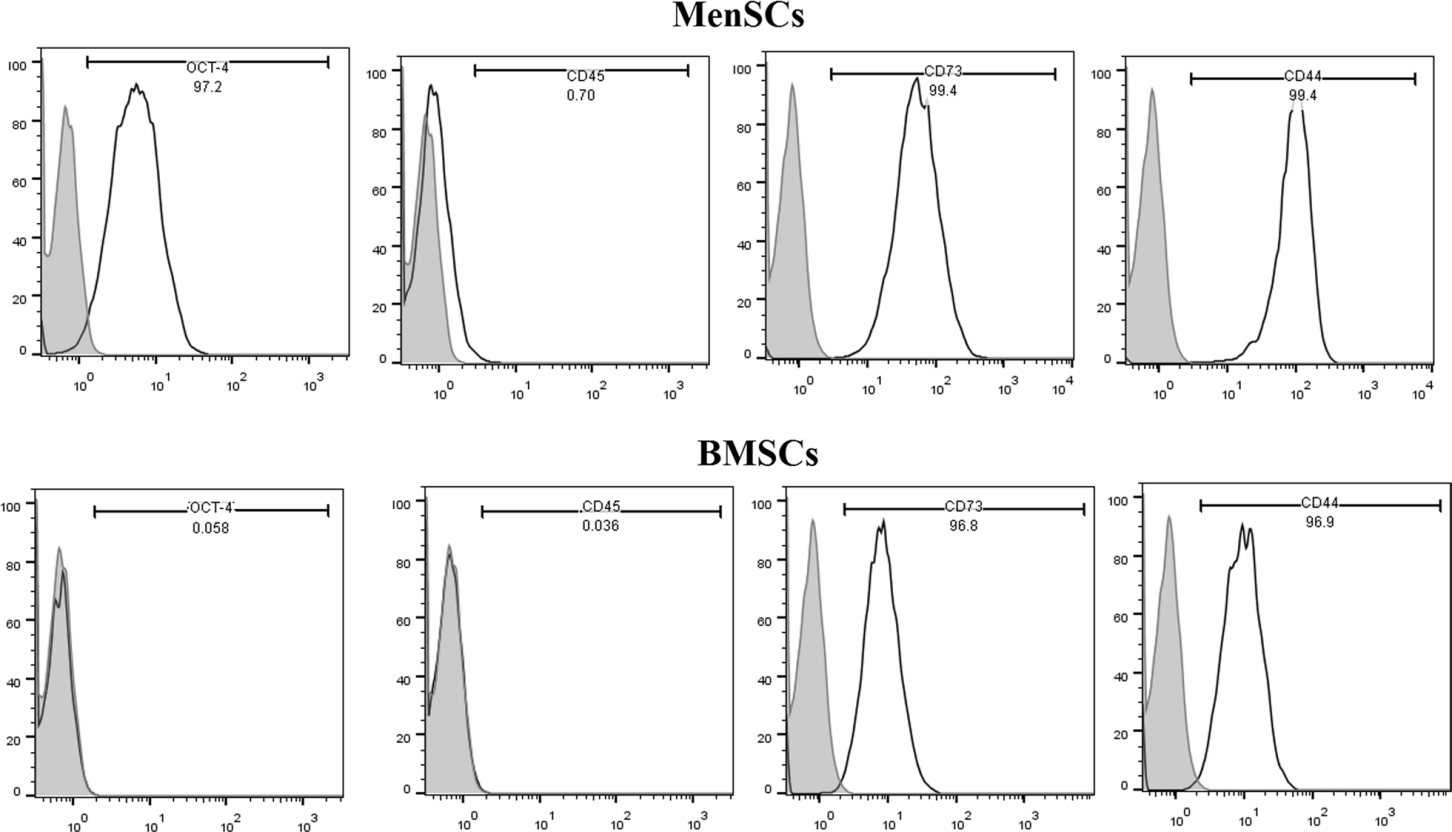
Flow cytometric analysis of MenSCs and BMSCs markers. Surface markers and their respective isotypes are shown as grey and black curves

Flow cytometry analysis was used to identify the surface markers expressed by MenSCs and BMSCs. MenSCs and BMSCs were highly expressed mesenchymal markers including CD73 and CD44, but negative for CD45 as hematopoietic marker. In contrast, only MenSCs expressed OCT-4 as the embryonic marker (Fig. 1).

### 5-aza and bFGF pre-treatment enhance the paracrine activity of MenSCs and BMSCs

**Fig. 2:**
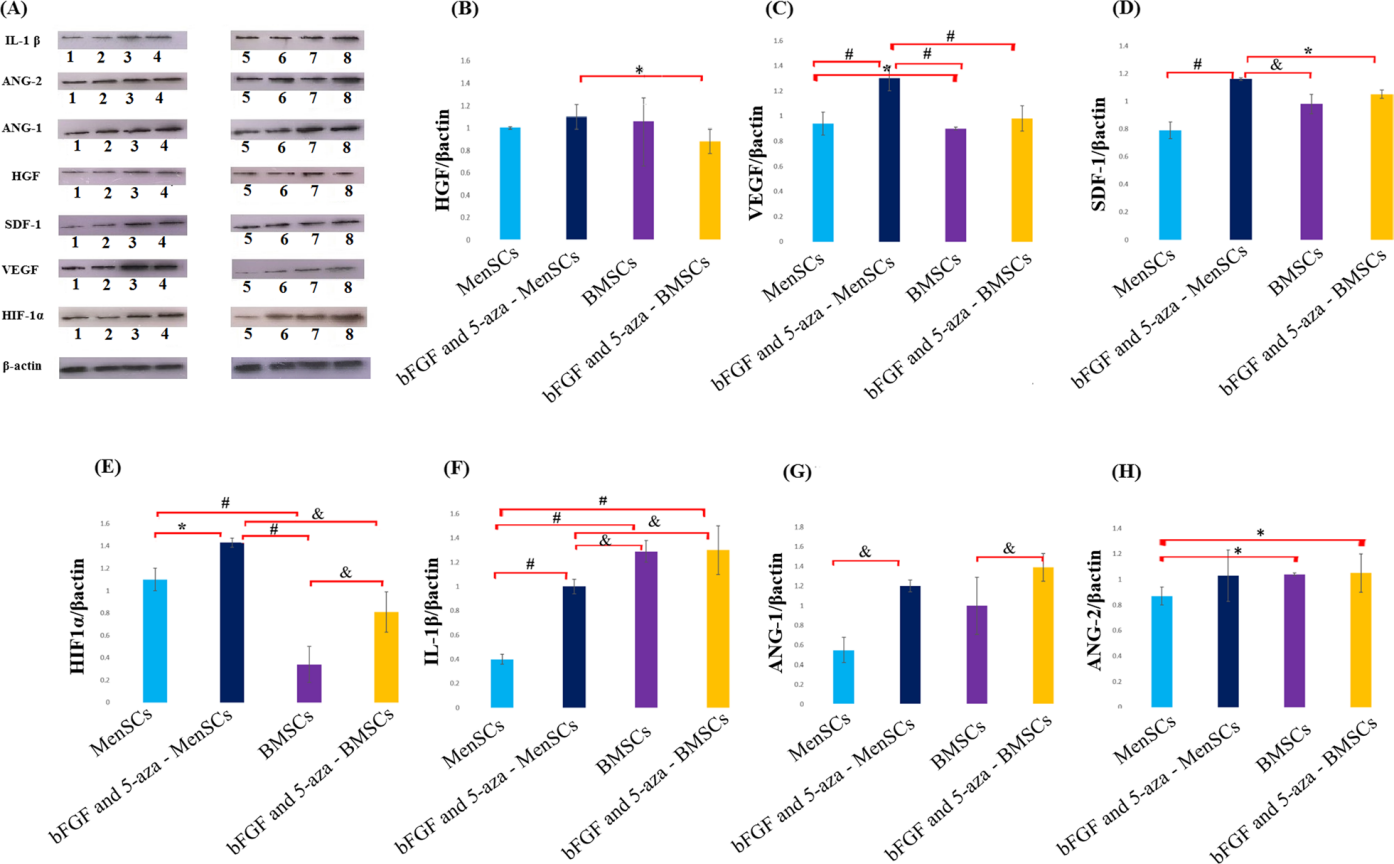
Effect of bFGF and 5-aza pre-treatment on VEGF, HIF α, SDF-1, ANG-1, ANG-2, IL-1 β and HGF proteins expression in MenSCs and BMSCs. **A** Western blotting analysis of SDF-1, VEGF, HGF, HIF-1α, IL-1 β, ANG-2, and ANG-1 expression. **B**–**H** Quantitative analysis of HGF, VEGF, SDF-1, HIF-1α, IL-1 β, ANG-2, and ANG-1. Two blots treated and compared exactly in the same conditions. Data are presented as means + SD. *P < 0.05, ^&^*P* < 0.01, and ^#^*P* < 0.001. 1,2: Before pre-treatment of MenSCs. 3, 4: After pre-treatment of MenSCs. 5,6: Before pre-treatment of BMSCs. 7,8: After pre-treatment of BMSCs

The levels of plasma interleukin and growth factors were quantified before and after 5-aza and bFGF pre-treatment. Result showed that pre-treatment with bFGF and 5-aza could significantly increase the level of VEGF, SDF-1, HIF-1α, IL-1β, and ANG-1 secretion from MenSCs (*P* < 0.001*, P* < 0.001*, P* < 0.05*, P* < 0.001 and *P* < 0.01 respectively); and HIF-1α and ANG-1 secretion from BMSCs (*P* < 0.01*,* and *P* < 0.01 respectively). Moreover, pre-treatment enhanced the secretion of VEGF, HIF-1α, and HGF from MenSCs in compare with BMSCs (*P* < 0.001*, P* < 0.01,* P* < 0.05 and *P* < 0.05 respectively). As Fig. 2 shows, the level of IL-1β secretion was significantly increased after bFGF and 5-aza pre-treatment of BMSCs in compared to MenSCs (*P* < 0.01). There were no significant differences in the levels of ANG-1 and ANG-2 after application of pre-treatment regimen on BMSCs and MenSCs (*P* ˃ 0.05) (Fig. 2, Additional file 1: Fig. S1).

### *bFGF and 5-aza pre-treated BMSCs and MenSCs improved cardiac function *in vivo

**Fig. 3:**
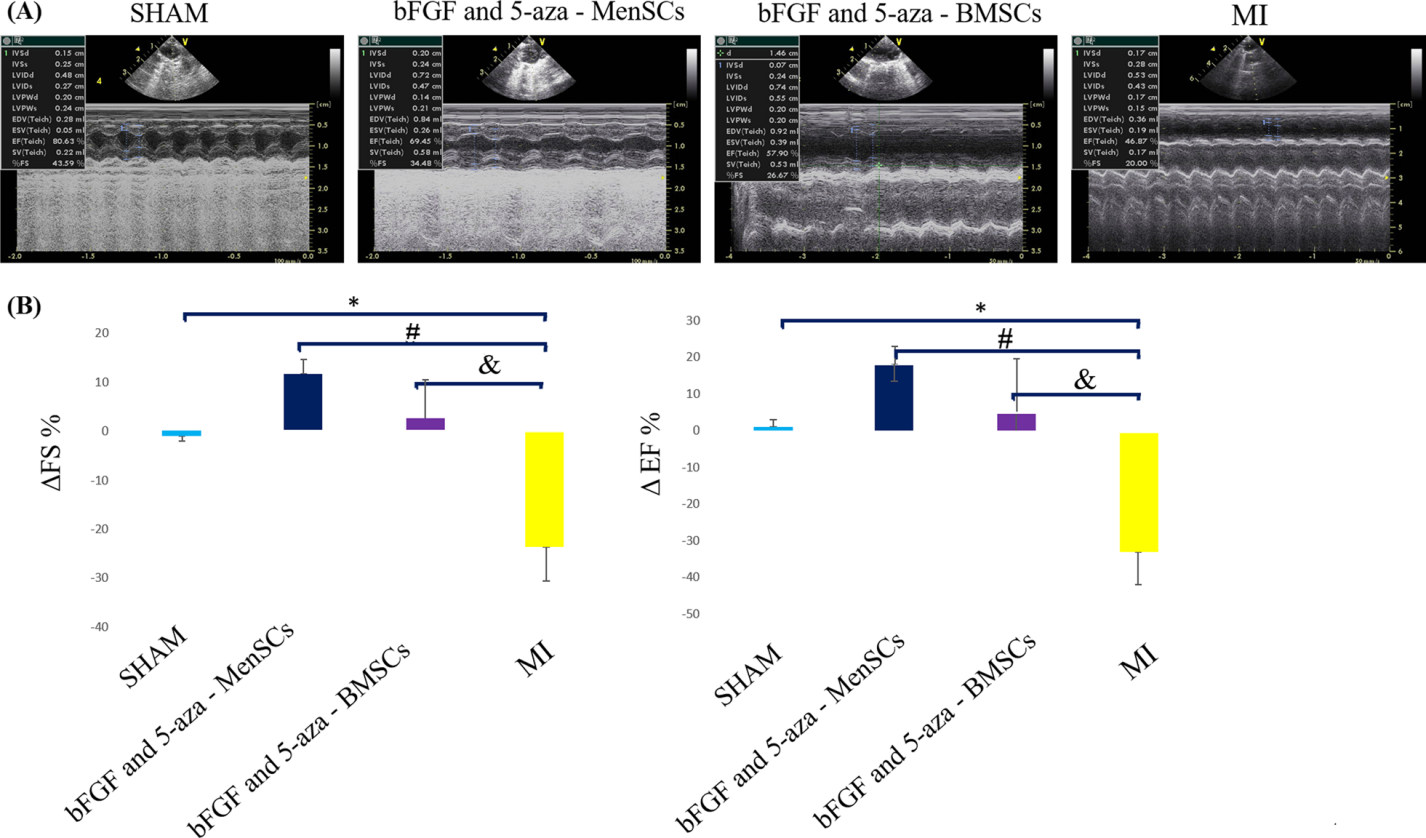
Evaluation of cardiac function after cell therapy by echocardiography. **A** Representative images of echocardiographic findings. **B** Echocardiography results revealed significant increases of ΔFS and ΔEF after bFGF and 5-aza pre-treated MenSCs and bFGF and 5-aza pre-treated BMSCs injection compare to the MI group. ^#^*P* < 0.001 and ^&^*P* < 0.01

On day 35 after surgery, the ΔEF and ΔFS were significantly greater in bFGF and 5-aza pre-treated stem cell applied groups than in the MI group. However, bFGF and 5-aza pre-treated MenSCs improved the ΔEF and ΔFS better than bFGF and 5-aza pre-treated BMSCs, there was no significant difference in ΔEF and ΔFS between these two groups (*P* *˃* 0.05) (Fig. 3).

### *bFGF and 5-aza pre-treated MenSCs downregulate the inflammatory responses *in vivo

The level of proinflammatory cytokines including IL‐1β, IL‐6, and TNF‐α in the serum was determined by ELISA. We observed that the expression levels of IL‐1β, TNF-α, and IL-6 were significantly upregulated in the MI group compared to the sham (*P* < *0.001, P* < *0.001,* and *P* < *0. 001* respectively*)*. ELISA analysis demonstrated that the expression of IL‐1β, TNF-α, and IL-6 were markedly enhanced in the MI group compared to the bFGF and 5-aza pre-treated MenSCs (*P* < *0.01, P* < *0.001,* and *P* < *0.001* respectively*)*. However, pre-treatment of MenSCs with bFGF and 5-aza significantly decreased the serum levels of IL-1β compared to the bFGF and 5-aza pre-treated BMSCs (*P* < *0.05*). There was no significant change in IL-1β concentration between the pre-treatment of BMSCs with bFGF and 5-aza group and the MI group (*P˃ 0.05*) (Table 1).

**Table 1:** The level of pro-inflammatory cytokines (IL-6, IL-1β, and TNF-α) in serum after systemic administration of bFGF, 5-aza pre-treated stem cells following MI

Groups	IL-6 (pg/mL)	TNF-α (pg/mL)	IL-1β (pg/mL)
Sham	77.55 ± 5	18.55 ± 2	80.35 ± 3
MI	240.10 ± 28^#^	85 ± 5^#^	443.55 ± 6^†&^
bFGF + 5-aza BMSCs	90.10 ± 6	23.2 ± 2	364.73 ± 2^†^*
bFGF + 5-aza MenSCs	78.20 ± 1	20.50 ± 1	186.60 ± 1

Data are presented as means + SD. * *P* < *0.05* vs MenSCs, ^&^*P* < *0.01* vs MenSCs, ^†^*P* < *0.001* vs sham group, and ^#^*P* < *0.001* vs MenSCs, BMSCs and sham group

### bFGF and 5-aza pre-treated MenSCs exert a protective role against myocardial infarction

**Fig. 4:**
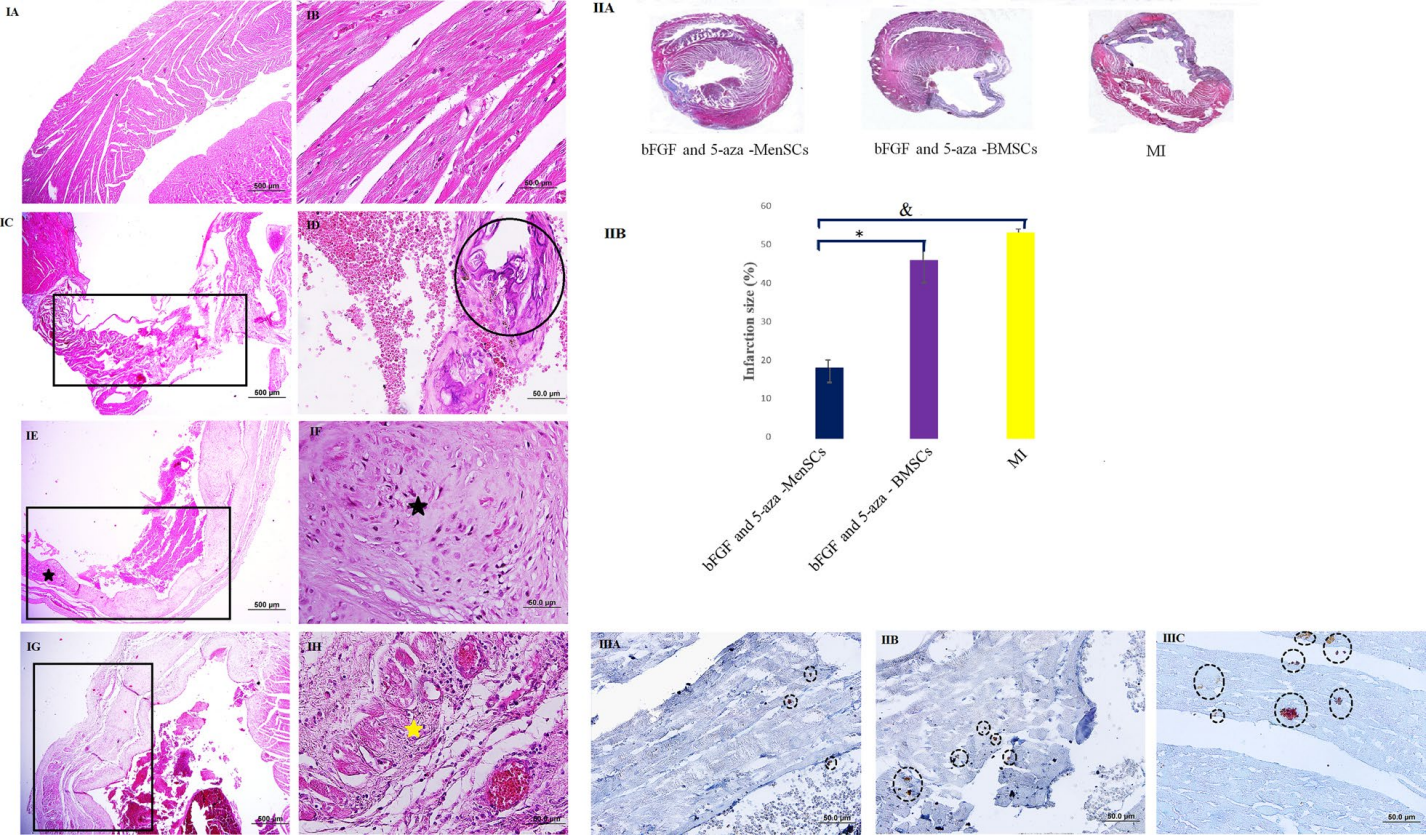
(**IA-H**): Representative heart sections stained with H&E. (**IA & IB**): Sham group, note to intact myocardial tissue, (**IC & ID**): MI group, (**IC**): Infarct area and progressive thinning of the ventricular wall is seen (rectangle), (**ID**): Note to endochondral ossification in infarct zone (circle), (**IE & IF**): bFGF and 5-aza pre-treated BMSCs received group, (**IE**): Noticeable thinning of the ventricular wall (rectangle) and cartilaginous metaplastic change (star) is evident, (**IF**): Higher magnification of the pervious slide, (**IG & IH**): bFGF and 5-aza pre-treated MenSCs received group, (**IG**): Note to the better preservation of the myocardial stature in infarct area (rectangle), (**IH**): Persevered cardiomyocytes (yellow star) are shown. (H & E, scale bar = IA, IC, IE, IG: 500 μm; IB, ID, IF, IH:100 μm). (**II**): Infarct size 35 days post-MI. (**IIA**): Representative pictures of left ventricle from each group after Masson's trichrome staining, (**IIB**): Infarct size is calculated from the ratio of the surface area of infarct wall and the entire surface area of the left ventricle. The data are expressed as the mean ± standard deviation, Scale bar: 1.5 mm. *P < 0.05, ^&^*P* < 0.01. (**III**): Tracking of stem cell engraftment after systemic administration in myocardium following MI using IHC, (**IIIA**): Only a limited number of human miothocondiria from BMSCc is detected in non-infarct zone, (**IIIB & IIIC**): Successful transfer of human mitochondria from MenSCs after systemic administration is seen in infarct zone and non-infarct zone respectively (IHC, Scale bar = 500 μm)

H&E stained sections belonged to the sham group demonstrated normal histological structure. In MI group, the cardiomyocytes were disrupted, and cell degeneration and death were apparent within the infarcted part. Decreased left ventricular wall thickness found in MI group as compared with the sham group. Also, there were cartilage formation and mineralization in sub endocardial areas. In group that bFGF and 5-aza pre-treated BMSCs were administrated seven days after induction of MI; degeneration of cardiomyocytes, necrotic myocardial cells, loss of muscle fiber integrity, dense scar formation, and also cartilaginous and/or osseous metaplastic changes were evident. On the other hand, bFGF and 5-aza pre-treated MenSCs alleviated MI-induced myocardial injury. There were noticeable restoration of the myocardial structure and dramatically smaller infarct site, with greatly less fibrosis and thinning of the LV wall. The myofilament abnormality was improved significantly (Fig. 4IA–H).

The mean myocardial infarct size in bFGF and 5-aza pre-treated MenSCs group (18.12 ± 5.33%) was less than that of the bFGF and 5-aza pre-treated BMSCs group (46.82 ± 8.33%), and MI group (53.46 ± 1.54%) (*P* < *0.05* and *P* < *0.01* respectively). No significant difference was detected between the bFGF and 5-aza pre-treated BMSCs and the MI group (*P* > *0.05*) (Fig. 4II).

To determine the degree of appropriated engraftment of the injected stem cells into the myocardium, the anti-human mitochondrial antibody which are ubiquitously and specifically expressed by human mitochondria was used. 28 days after systemic administration of stem cells, the homing of these cells into the myocardium was detected. Limited number of human mitochondria from BMSCs were still detectable in the non-infarct myocardium, and human mitochondria from these cells did not integrate appropriately with the cardiomyocytes in infarct site. In other hand, human mitochondria from MenSCs group was detected in infarct site and border zone, furthermore appropriate integration of this organelle with cardiomyocytes and endothelial cells were also seen (Fig. 4III).

### bFGF and 5-aza pre-treated MenSCs decrees the level of NF-κB expression

**Fig. 5:**
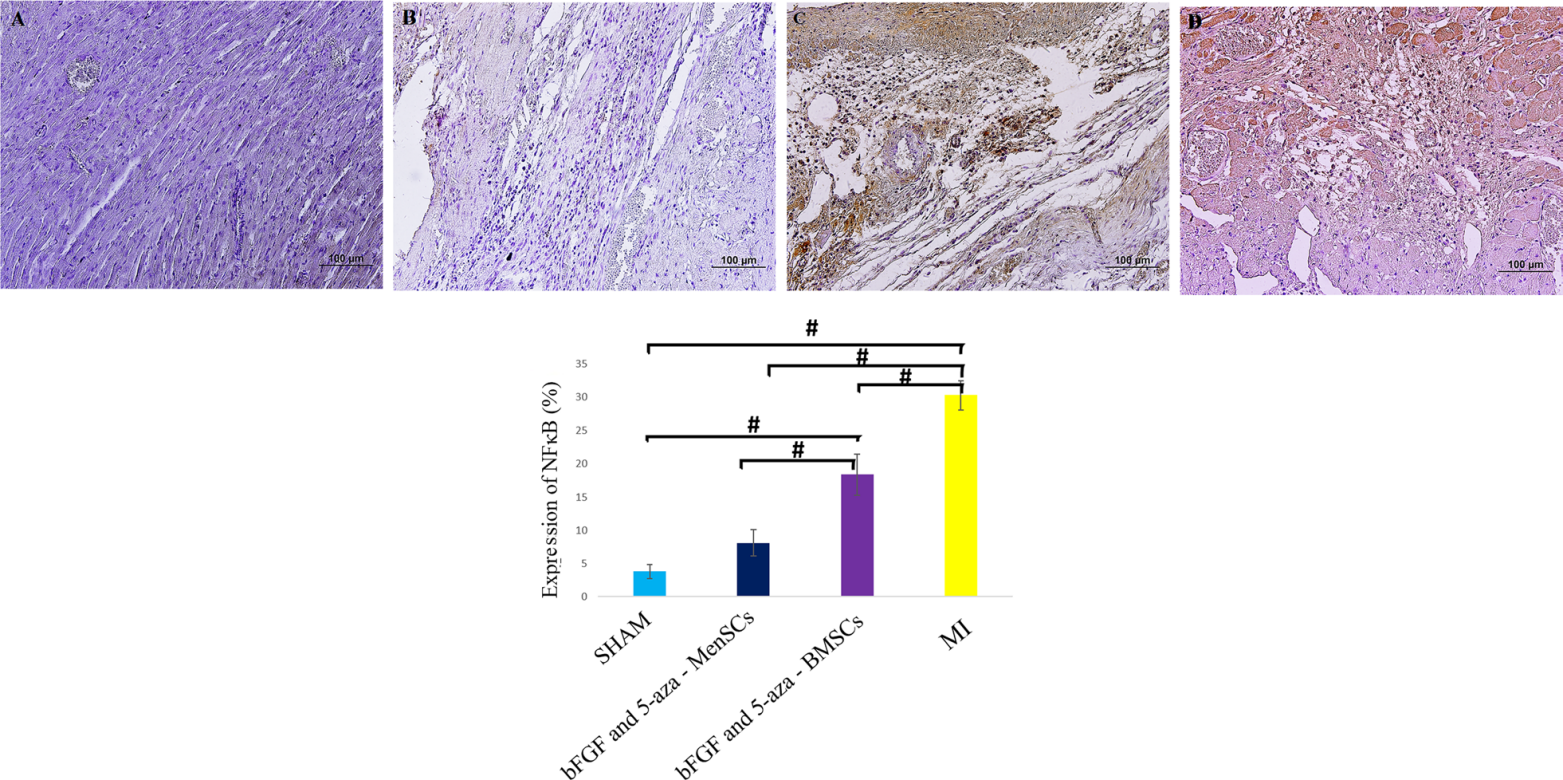
IHC staining for detection of NF-κB expression in myocardium **A** Sham, **B** bFGF and 5-aza pre-treated MenSCs received group, **C** bFGF and 5-aza pre-treated BMSCs received group, **D** MI group. Brown colour indicates NF-kB positivity (IHC, Scale bars = 100 µm)

28 days after the stem cell administration, NF-κB expression was compared in all groups by IHC assay. We found that bFGF and 5-aza pre-treated MenSCs more efficiently diminish the NF-κB activity compared with bFGF and 5-aza pre-treated BMSCs in cardiac tissue (*P* < *0.001*) (Fig. 5).

### bFGF and 5-aza pre-treated BMSCs and MenSCs promote angiogenesis after MI

**Fig. 6:**
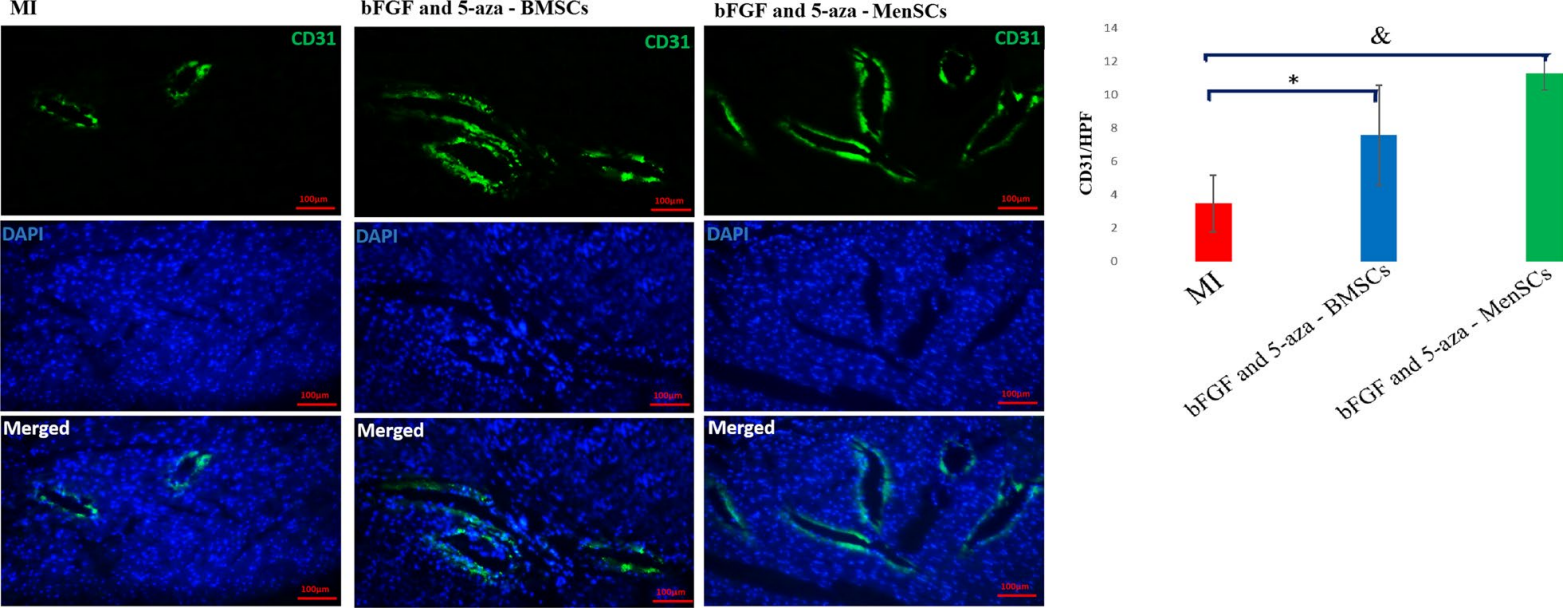
Myocardial angiogenesis. **A** Immunofluorescence staining for endothelium (CD31, in green), and nuclei (DAPI, in blue) in sections of myocardium following systemic administration of pre-treated stem cells. **B** Quantitation of angiogenesis in pre infarct zone, (Scale bar = 100 µm *P < 0.05, and ^&^*P* < 0.01)

Vascular density at peri-infarct border zone border zone were calculated based on endothelial-cell marker. CD31 + vessel density was considerably greater in sections from the hearts of the pre-treated stem cell administrated groups compared to the control MI group (Fig. 6).

## Discussion

MI as one of the serious causes of global mortality and is associated with dysfunction and irreversible loss of cardiomyocytes. Cell therapy exerts its therapeutic effects through different mechanisms such as angiogenesis, anti-apoptosis of cardiomyocytes, and anti-inflammation [31]. To obtain good outcomes in stem cell therapy, it is critical to prepare stem cells with a high therapeutic potential for transplantation. The synergic effect of 5-Aza and bFGF in MSCs confirmed through a high expression of TNNT1, Desmin, and NKX2.5 as the cardiac-specific biomarkers [32].

5-aza treatment could increase the immunosuppressive effects of MSCs by the promotion of secretion of immunomodulatory factors from MSCs [19]. We previously reported that bFGF and 5-aza stimulation could upregulated cardiac markers in MenSCs in compared to BMSCs [33]. In the present study, we evaluated the therapeutic efficacy of systemic administration of bFGF and 5-aza pre-treated BMSCs and MenSCs in improving myocardial repair in the rat MI model. We found that synergic application of bFGF and 5-aza upturn the paracrine effect of MenSCs compare to BMSCs and pre-treatment of MenSCs with bFGF and 5-aza could considerably protect myocardial tissue from subsequent injuries following MI with various mechanisms including: preservation of cardiomyocytes form death, altitude of angiogenesis, and prevention of fibrosis progress. Also, bFGF and 5-aza pre-treated MenSCs more efficiently exerted immunomodulatory effects in compared to BMSCs. Indeed, our results have shown that compare to bFGF and 5-aza pre-treated BMSCs, bFGF and 5-aza pre-treated MenSCs robustly induced the expression of VEGF, HIF-1α, and HGF, which are decisive for angiogenesis. Also bFGF and 5-aza pre-treated MenSCs administration could noticeably increase the blood vessel density in the peri-infarct area in rat model. Angiogenesis is an important mechanism for MI therapy; studies have shown that angiogenesis can improve cardiac function in infarcted hearts [34]. The increased vascular density in the experimental group transplanted with bFGF and 5-aza pre-treated MenSCs maybe lead to enhancement of preserved cardiomyocytes and ventricular contractile functions. Furthermore, we have demonstrated that bFGF and 5-aza pre-treated MenSCs significantly enhanced the expression of VEGF, HGF, and HIF-1α. HGF as an angiogenic factor initiate angiogenesis [14]. On the other hand, HIF-1α is the main transcriptional factor of VEGF under hypoxic condition [35]. HGF can promote the expression of VEGF by induction of HIF-1α expression and also, HGF induces HIF-1α expression and subsequent processes to promote the expression of VEGF [16].

A key factor for the success of stem cell therapy is the potential of stem cells for homing to the injury site to exert their beneficial effects. SDF-1 plays a pivotal role in the stem cell homing [17]. In the present study, pre-treatment with bFGF and 5-aza increased secretion of SDF-1 in MenSCs more significantly after pre-treatment than BMSCs. Elevated SDF‑1 expression in the conditioned medium following pre-treatment with bFGF and 5-aza,could increase C‑X‑C chemokine receptor type 4 (CXCR‑4) on the surface of MenSCs that can lead to efficacious migration of stem cells to the site of infarction and/or ischemic myocardium [36]. In this study, although bFGF and 5-aza pre-treated BMSCs and MenSCs were injected via the tail vein, we have found that BMSCs and MenSCs migrated to the infarcted area without any rejection by the recipients. Studies reported that despite trapping in other organs post Intravenous injection, stem cells are able to homing specifically in the infarcted myocardium in response to injury signals that produce infarction area [37, 38]. Min et al. have shown that some cytokines, which are released locally in injured myocardium such as TNFα, play important role in homing of stem cells [39]. Studies have also shown that the increase of SDF-1 can lead to immunosuppression. Xiang Li et al. indicated that SDF-1 mediates the MenSCs’ immunomodulatory effects by reducing the level of dendritic cell population, stimulated both type 2 macrophages and regulatory T cells [36]. Thus, pre-treatment with bFGF and 5-aza enhance the expression of SDF-1 which most plausibly is one of the factors underlying its increasing effects on MenSCs migratory potential and decrease fibrosis.

We demonstrated that compared with the bFGF and 5-aza pre-treated BMSCs, the expression of IL-1β was considerably lower in pre-treated MenSCs. The present study also demonstrated that bFGF and 5-aza pre-treated MenSCs more effectively, could inhibit NF-κB expression in cardiac tissue. The death of cardiomyocytes can promote the expression of inflammatory cytokine such as IL-1β by NF-κB pathways [40]. Inflammatory cytokines trigger the characteristic of cardiac remodelling such as replacement of the infarcted area by scar, myocyte hypertrophy, myocyte loss through apoptosis, alterations of the extracellular matrix, and eventually cardiac dysfunction [41]. Reduced inflammation may be one reason attenuated myocardial fibrosis in bFGF and 5-aza MenSCs group than bFGF and 5-aza BMSCs group.

Despite encouraging results, there were some limitations in this study. First, further investigation is required to determine how bFGF and 5-aza increase the paracrine activity of MenSCs. Second, the optimal dose and time of bFGF and 5-aza application with regards to the sources of stem cells should be investigated. Finally, the distinct paracrine factors of bFGF and 5-aza pre-treated MenSCs and bFGF and 5-aza pre-treated BMSCs have not been fully determined.

## Conclusion

Our research demonstrated that bFGF and 5-aza pre-treated MenSCs offer superior cardioprotection compare to bFGF and 5-aza pre-treated BMSCs following MI. The protective effect of bFGF and 5-aza pre-treated MenSCs is associated with paracrine effects that are involved in angiogenesis and cardiac function. Also, these results showed that injection of the pre-treatment of MenSCs with bFGF and 5-aza had anti‐inflammatory effects in the cardiac tissue which was indicated by the lower release of proinflammatory cytokines and reduced NF-κB expression. These results illustrate that bFGF and 5-aza pre-treating would be considered an effective factor to improve the biological functions of MenSCs, especially via angiogenesis and immunomodulatory effects.

## Supplementary Information

**Additional file 1**. Raw data of western blot.

## Data Availability

The datasets used and/or analysed during the current study are available from the corresponding author on reasonable request.

## Abbreviations

HF: Heart failure
MI: Myocardial infarction
BMSCs: Bone marrow stem cells
MB: Menstrual blood
MenSCs: Menstrual blood stem cells
MSCs: Mesenchymal stem cells
ANG-2: Angiopoietin 2
VEGF: Vascular endothelial growth factor
HGF: Hepatocyte growth factor
MMPs: Matrix metalloproteases
FGF: Fibroblast growth factor
CM: Conditioned medium
5-aza: 5-Aza-2ʹ-deoxycytidine
bFGF: Basic fibroblast growth factor
EDTA: Ethylenediaminetetra acetic acid
PBS: Phosphate buffered saline
DMEM-F12: Dulbecco’s modified eagle’s medium/f12
HIF-1: Hypoxia-inducible factor 1
IL-1β: Interleukin 1 beta
SDF-1: Stromal cell-derived factor-1
PVDF: Polyvinylidene difluoride
LAD: Left anterior descending
FS: Fractional shortening
EF: Ejection fraction
LVIDd: Left ventricle internal diameter in diastole
LVIDs: Left ventricle internal diameter in systole
NBF: Neutral buffered formalin
H&E: Haematoxylin and eosin
LV: Left ventricular
TBS: Tris-buffered saline
HPF: High-power fields
IL-6: Interleukin (IL)-6
TNF-α: Tumor necrosis factor alpha
DAB: 3, 3’-Diaminobenzidine
ELISA: Enzyme-linked immunosorbent assay
CXCR-4: C-X-C chemokine receptor type 4
