# Supplementary figures and images for: Greater angiogenic and immunoregulatory potency of bFGF and 5-aza-2ʹ-deoxycytidine pre-treated menstrual blood stem cells in compare to bone marrow stem cells in rat model of myocardial infarction
Source: BMC Cardiovasc Disord. 2022 Dec 31;22:578. doi: 10.1186/s12872-022-03032-7 (PMC9805241; doi:10.1186/s12872-022-03032-7)

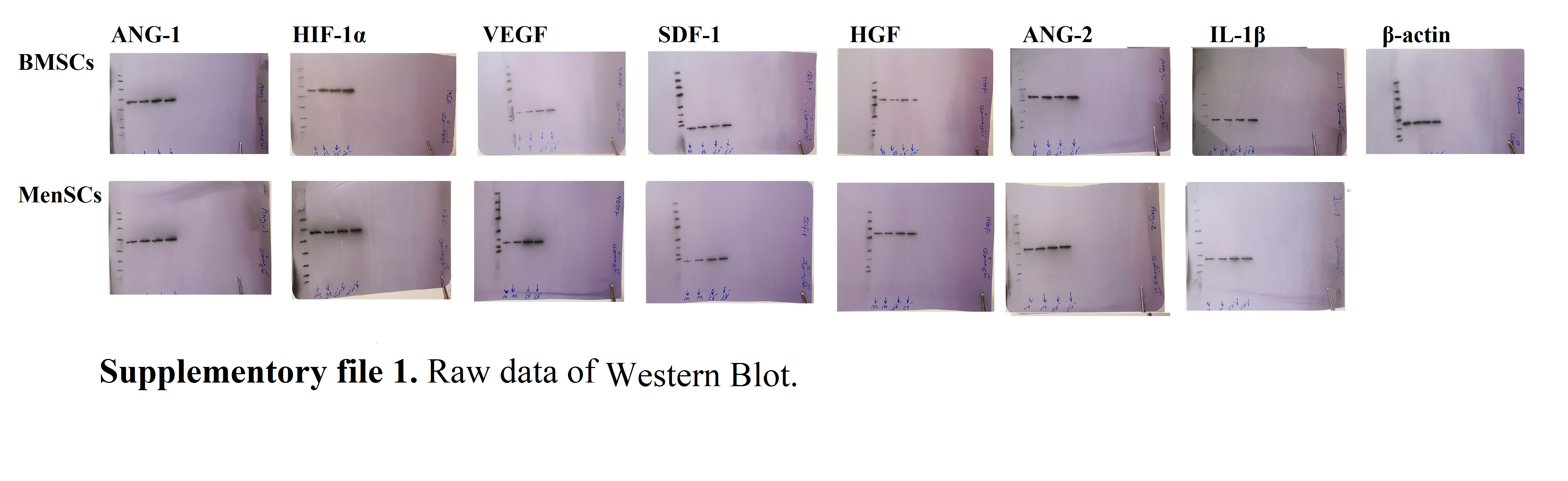

Supplement: Supplementary file 1 — Additional file 1. Raw data of western blot. [file 12872_2022_3032_MOESM1_ESM.png]
